# CAREGIVERS & COLONOSCOPIES: A QUANTITATIVE COMPARATIVE STUDY OF CAREGIVERS’ PREVENTATIVE HEALTH BEHAVIORS

**DOI:** 10.1093/geroni/igad104.3211

**Published:** 2023-12-21

**Authors:** Claire Quinlan, Samantha Brady, Sophia Ashebir, Alexa Balmuth, Lisa D’Ambrosio, Joseph Coughlin

**Affiliations:** Harvard Medical School, Boston, Massachusetts, United States; Massachusetts Institute of Technology, Cambridge, Massachusetts, United States; Massachusetts Institute of Technology, Cambridge, Massachusetts, United States; Massachusetts Institute of Technology, Cambridge, Massachusetts, United States; Massachusetts Institute of Technology, Cambridge, Massachusetts, United States; Massachusetts Institute of Technology, Cambridge, Massachusetts, United States

## Abstract

That many caregivers experience some degree of burden is well-established, but little research has investigated the association of burden on caregiver preventative health behaviors. A lack of adherence to these behaviors, including to recommended colonoscopy and mammography screenings, routine vaccinations, annual clinic visits, and HIV testing, may portend worsening health outcomes for family caregivers if diseases are left undetected or untreated; such outcomes could also be caused by and contribute to the crisis of lack of care to meet demand. Survey questionnaires (N=326) were completed by 214 current caregivers and 112 former caregivers from the national MIT AgeLab Caregiver Panel. Respondents predominately identified as white (88%) and female (83%), with a median age of 40-49 years old. Questions assessed caregiving intensity, health status, reported caregiving burden, and adherence to preventative health screenings and vaccinations (per USPTF age- and gender-based recommendations). Among all caregivers, just 17% reported that they were up-to-date with appropriate preventative health screenings. Current caregivers were less likely than previous caregivers to be up-to-date. Controlling for age, income level, and health status, caregiving intensity (as measured by hours per week) was an independent predictor of adherence to preventative health screenings. These findings suggest that current caregivers who are providing high-intensity care are less likely to be engaging in preventative health behaviors for themselves. This finding is concerning for the future health of these caregivers as well as their care recipients and highlights the need for primary care preventative health initiatives specifically targeting family caregivers.

